# Hydroxychavicol‐Rich *Piper betle* Extracts Attenuate TNFα‐Driven Endothelial Inflammatory Response by Modulating Adhesion Molecules and Cytokine Release

**DOI:** 10.1155/adpp/7056593

**Published:** 2026-11-03

**Authors:** Siti Marliana Jasni, Yiizamy Suffian, Nor Hisam Zamakshshari, Erna-Zulaikha Dayang

**Affiliations:** ^1^ Department of Paraclinical Sciences Faculty of Medicine and Health Sciences Universiti Malaysia Sarawak 94300 Kota Samarahan Sarawak Malaysia unimas.my; ^2^ Department of Chemistry Faculty of Resource Science and Technology Universiti Malaysia Sarawak 94300 Kota Samarahan Sarawak Malaysia unimas.my

**Keywords:** adhesion molecules, atherosclerosis, cytokines, inflammation, *Piper betle*, TNF-α

## Abstract

Atherosclerosis is a chronic inflammatory disorder that involves endothelial dysfunction and accumulation of inflammatory cells in the vessel intima. Approximately 32% of global mortality is attributed to cardiovascular diseases, with atherosclerosis playing a major role. Endothelial dysfunction is a key hallmark of atherosclerosis, with key mediator tumor necrosis factor‐α (TNF‐α) driving endothelial–monocyte interactions that further lead to adverse cardiovascular events. Hydroxychavicol, a phenolic compound enriched in *Piper betle* (PB), methanolic and chloroform extracts, has demonstrated anti‐inflammatory activity, yet its effects on endothelial activation remain insufficiently characterized. Therefore, this study aimed to investigate the endothelial‐protective effects of hydroxychavicol‐rich PB methanol (PB‐M) and chloroform (PB‐C) extracts by evaluating their impact on the expression of adhesion molecules and proinflammatory cytokines in TNF‐α‐induced human umbilical vein endothelial cells (HUVECs). HPLC was performed to confirm the presence and determine the amount of hydroxychavicol in both PB‐M and PB‐C. The cytotoxicity effects of both extracts were first assessed by MTT assay. HUVECs were pretreated with NF‐κB inhibitor BAY11‐7082, PB‐M, or PB‐C before being exposed to TNF‐α for 4 h. Adhesion molecules and cytokines expression were measured by Western blot and ELISA, respectively. Both PB‐M and PB‐C were shown to contain hydroxychavicol, yet PB‐C has about 3.8 times higher levels. MTT assays yielded IC_50_ values of 128.9 µg/mL for PB‐M and 18.59 µg/mL for PB‐C, while HUVEC morphology remained normal at 100 µg/mL PB‐M and 20 µg/mL PB‐C. Both extracts displayed dose‐dependent suppressing effects on TNF‐α‐induced E‐selectin, VCAM‐1, and ICAM‐1 expression. PB‐M resulted in a pronounced inhibition of IL‐6, IL‐8, and MCP‐1, while PB‐C displayed cytokine‐selective suppressive effects. In conclusion, both PB‐M and PB‐C attenuated TNF‐α‐induced endothelial inflammation, with PB‐C acting at lower effective doses but PB‐M providing broader anti‐inflammatory suppression, suggesting that their different phytochemical compositions and possible synergistic interactions beyond hydroxychavicol may contribute to their distinct bioactivities, warranting further investigations.

## 1. Introduction

Atherosclerosis is a chronic inflammatory disease characterized by persistent inflammation of vascular endothelial cells and the intima layer of large‐ and medium‐sized arteries, leading to an accumulation of inflammatory cells and lipid within blood vessels [1]. Globally, approximately 32% of deaths in 2022 were attributed to cardiovascular diseases, including atherosclerosis [2]. Multiple bodies of evidence suggested that endothelial dysfunction is a hallmark of atherosclerotic vascular disease [3]. Although statins, a standard treatment for atherosclerosis, help restore endothelial function and slow disease progression [4], their use is increasingly linked to side effects such as Type 2 diabetes and musculoskeletal pain [5, 6].

Tumor necrosis factor‐α (TNF‐α) is pivotal in the mediation of inflammatory responses, including those contributing to the pathogenesis of cardiovascular disease [7]. TNF‐α is a well‐established mediator used in previous studies of endothelial dysfunction [8, 9], consistent with its established involvement in atherogenesis. Elevated TNF‐α was shown to be associated with an increased risk of ischemic events [10]. At the molecular level, the interaction of TNF‐α and TNF‐α receptor 1 (TNFR‐1) on the surface of vascular endothelial cells initiates intracellular signaling cascades, including the activation of the NF‐κB pathway [11, 12]. To this end, the activation of the NF‐κB pathway results in the expression of adhesion molecules, namely, E‐selectin, VCAM‐1, and ICAM‐1 [13, 14]. Furthermore, activated endothelial cells actively respond to inflammation by endogenously producing various proinflammatory cytokines and chemokines, such as IL‐6, IL‐8 and MCP‐1, which enhance communication between endothelial cells and attract immunological cells in the circulatory system [15]. Collectively, these molecules facilitate monocyte recruitment and transmigration across endothelial cells, enhancing foam cell formation and plaque formation, thereby accelerating the disease progression [16].

*Piper betle* (PB), locally known as “sirih” or betel vine, is a member of the Piperaceae family with over 1000 species of *Piper* spread worldwide and over 400 species reported in Malaysia alone. Among the *Piper* species found in Malaysia are *P. abbreviatum, P. aborescens, P. betle, P. caninum, P. duncum, P. erecticaule, P. lanatum, P. miniatum, P. majusculum, P. nigum, P. porphyrophyllum, P. ribesioides, P. stylosum,* and *P. umbellatum* [17–19]. Traditionally, PB is known for its therapeutic purposes, especially anti‐inflammatory and antibacterial [20]. Previous in vivo studies have revealed that PB methanol (PB‐M) extracts exhibit anti‐inflammatory properties in disease models such as rheumatoid arthritis and gout [21, 22]. Additionally, a previous study conducted in vitro has demonstrated the anti‐inflammatory effect of PB‐M extracts in RAW 264.7 macrophages [23].

Extraction of plant materials is commonly performed using solvents of different polarities to solubilize various phytochemicals based on their polarity [24]. Accordingly, the present study employed methanol and chloroform as extraction solvents. Whilst the findings in these studies expand our understanding of the anti‐inflammatory potential of PB, the effects of PB extracts of different polarities, i.e., methanol and chloroform extracts, on TNF‐α‐induced expression of adhesion molecules in endothelial cells remain limited. Both chloroform and methanol extracts of PB contain secondary metabolites such as hydroxychavicol [25], a potent anti‐inflammatory compound against macrophage and T cells [23, 26], yet it remains unclear which extract is more effective at mitigating endothelial inflammatory responses. Therefore, this study aimed to compare the endothelial‐protective effects of PB methanol (PB‐M) and PB‐C extracts on the expression of TNF‐α‐induced inflammatory adhesion molecules, namely, E‐selectin, VCAM‐1 and ICAM‐1, and proinflammatory cytokines involved in leukocyte recruitment, namely, IL‐6, IL‐8, and MCP‐1. The findings of this study could provide new insight into the possible application of PB extracts in mitigating endothelial activation associated with inflammatory diseases.

## 2. Materials and Methods

### 2.1. Plant Sampling and Extract Preparation

Three kilograms of PB leaves (voucher specimen no. UNIMASNHZ002) were collected between 6 December 2024 until 22 December 2024 from Kampung Belangkan, Rantau, Negeri Sembilan, Malaysia (2.653428°N, 101.938784° E). The leaves were dried for two weeks at room temperature until constant weight was achieved. Then, the dried leaves were pulverized into fine powder (982 g) and subsequently soaked in chloroform for 72 h, a process that was repeated three times consecutively. The yielded PB‐C extracts were combined, filtered through filter paper and concentrated using a rotary evaporator. For PB‐M, a separate batch of PB leaves was independently dried and pulverized as described above and subsequently extracted with methanol following the same extraction procedure.

### 2.2. Quantitative Analysis of Hydroxychavicol in PB Extracts Using HPLC

Quantitative analysis was performed using HPLC to determine the amount of hydroxychavicol based on a previous report with slight modification [25]. HPLC was performed using Shimazu reversed‐phase on C‐18 column (250 mm x 4.6, µm) and UV detection at 280 nm. The sample was run under an isocratic system with solvent A consisting of methanol (70%) and solvent B consisting of water (30%) for 15 min. Twenty µL of sample and standard hydroxychavicol were injected into the HPLC column at a flow rate of 0.7 mL/min. Hydroxychavicol was identified by comparing the retention periods of the sample with the standard on the HPLC chromatogram. The multipoint calibration curve approach was used to create a calibration curve. Twenty µL of pure hydroxychavicol concentrations (1–0.0625 mg/mL) were prepared and injected into the HPLC. A calibration curve was obtained by plotting a graph of concentration against the area under the curve.

### 2.3. Human Umbilical Vein Endothelial Cells (HUVECs) Cultivation

HUVECs (cat no. C‐12203, Promocell, GmBH, Germany) were cultured in the Pharmacology and Medical Molecular Genetic Laboratory, Faculty of Medicine and Health Sciences, UNIMAS, at 37°C with 5% CO_2_/95% air conditions. HUVECs were supplemented with EGM medium with the addition of 1% penicillin–streptomycin (1% v/v). The cell passage from passages between 5 and 8 were used in all experiments.

### 2.4. Cytotoxicity of PB Extracts on Endothelial Cells

The cytotoxicity of PB extracts in endothelial cells was determined by MTT assay (cat no. 11465007001, Sigma Aldrich, Germany) in accordance with the manufacturer’s guidelines. HUVECs were seeded in 96‐well plates with the density of 10,000 cells/well. The cytotoxic effects of PB‐M and PB‐C were evaluated at five increasing concentrations (1–150 µg/mL and 1–100 µg/mL, respectively). Different concentrations of the extracts were added into the designated wells, and the cells were incubated for another 24 h. Subsequently, MTT reagent and, after 4 h, solubilized buffer was added. The absorbance was measured at 600 nm using a microplate reader after an overnight incubation with solubilized buffer (Biotek Cytation 5, Agilent Technologies, USA).

### 2.5. Cell Treatment and Protein Sample Preparation

The fully confluent HUVEC monolayer were pretreated for 30 min with three different PB extracts concentration predetermined from the cytotoxicity assay (PB‐M: 1, 10, and 100 µg/mL; PB‐C: 1, 10, and 20 µg/mL). Ten µM of IκB inhibitor, BAY 11‐7082 (cat no. HY‐13453, MedChemExpress, USA), was used as a standard drug [27]. After 30 min, endothelial cells were stimulated with TNF‐α at 100 ng/mL (cat no. H8916, Sigma‐Aldrich, USA) in the presence or absence of the standard drug and extracts for 4 h. Cells treated with TNF‐α alone served as a negative control. Medium was collected and centrifuged at 12,000 rpm at 4°C. The supernatants were collected and kept at −20°C until subsequent ELISA analyses. HUVECs were washed with ice‐cold phosphate‐buffered saline (PBS) and lysed in 100 µL of radioimmunoprecipitation assay buffer (RIPA) (cat no. 89900, Thermo Scientific, USA). The protein lysate was centrifuged for 10 min at 12,000 rpm at 4°C. The supernatants were kept at −20°C until further analyses.

### 2.6. Protein Concentration Determination

The protein concentration from the cell lysates was determined by the Bicinchoninic Acid assay (BCA assay). For the standard curve of the assay, Pierce bovine serum albumin (BSA) stock 2 mg/mL (Thermo Scientific, USA) was diluted to achieve the concentrations of 200, 100, 50, 25, 12.5, and 6.25 µg/mL, respectively. The protein samples were diluted to 10X, 20X, and 40X, respectively, using deionized water. The Pierce BCA Protein Assay Kit (cat no. XD340640, Thermo Scientific, USA) working reagent was prepared according to the manufacturer’s guidelines by mixing Reagent A and Reagent B at the ratio of 1:50. Then, 200 µL of working reagent were added into 25 µL of protein samples in the 96‐well plate and incubated at 37°C for 30 min before measuring the absorbance at 562 nm using Biotek Cytation 5 (Agilent Technologies).

### 2.7. Western Blot

Approximately 8 µg of protein samples were loaded into 10% polyacrylamide gels. The protein separation was done via electrophoresis technique for 1.5 h (70V through stacking gel and 170V through running gel). Subsequently, protein was transferred onto the nitrocellulose membrane (Bio‐Rad Laboratories, California, USA) for 2 h at 0.22A. The presence of protein bands transferred on the membrane was determined via Ponceau staining.

Immunostaining was performed to detect the presence of targeted adhesion molecules and housekeeping protein using human anti‐rabbit E‐selectin (cat no. PA5‐29946, Invitrogen, USA), VCAM‐1 (cat no. 13662S, Cell Signalling Technologies, USA), ICAM‐1 (cat no. NBP1‐88700, Novus Biological, USA), and GAPDH (cat no. NB100‐56785, Novus Biological, USA) antibodies. Goat anti‐rabbit secondary antibody (cat no. 4030‐05, Southern Biotech, USA) was used in the subsequent incubation protocol. Both primary antibodies and secondary antibodies were prepared with the dilution factor of 1:5000 in 5% BSA (cat no. V900933, Sigma Aldrich, USA) in TBST. Blots were incubated in primary antibody diluent and subsequently in secondary antibody diluent for 1 h at room temperature with three 10‐min‐long washes in between. The protein bands were detected using Super Signal West Pico PLUS Chemiluminescent Substrate (cat no. 34080, Thermofisher Scientific, USA) and a chemiluminescence imaging reader, Amersham 680 (GE Healthcare, USA). The semi‐quantification of the protein band was performed via ImageJ software.

### 2.8. ELISA

ELISA kits were used to measure human IL‐6 (cat no E‐EL‐H6156, Elabscience, China), IL‐8 (cat no. E‐EL‐H6008, Elabscience), and MCP‐1 (cat no. EH0222, FineTest, China) in the medium supernatants of each sample. Briefly, a monoclonal antibody‐specific precoated plate was filled with 100‐µL diluent‐diluted samples. After 1 h of room temperature incubation with cytokine‐specific enzyme‐linked polyclonal antibody, the microtiter plate was washed 3 times. Ninety µL of substrate solution were added to each well after 5 washes, and the plate was kept out of the light for 15 min of room temperature incubation. Fifty µL of stop solution were added to stop the reaction, and the absorbance was measured at 450 nm.

### 2.9. Statistical Analyses

One‐way ANOVA with Bonferroni multiple comparison statistical tests was conducted using GraphPad Prism Version 10.4 software. The mean ± standard error of mean (SEM) from three biological replicates was used to report all results. *p* < 0.05 was considered statistically significant.

## 3. Results

### 3.1. HPLC Reveals That Hydroxychavicol Content is Higher in PB‐C Than in PB‐M

In this study, the percentage yields of PB‐C and PB‐M were 7.56% and 19.10%, respectively. The percentage yield was calculated using the following equation:

(1) Yield%=Dry extractgRaw materialsg×100.

Five different standards of hydroxychavicol were run on the HPLC, and the result was plotted. The standard graph for hydroxychavicol was obtained from the calibration equation as follows:

(2) y=3×107x−2173810.9992,r2=,

where *y* is the peak area and *x* is the weight of hydroxychavicol content in the extract (Figure 1A). Hydroxychavicol appeared at the retention time of 4.3 min of the HPLC spectrum (Figure 1B). The amounts of hydroxychavicol in the chloroform and methanol extracts were 0.14462 and 0.03781 mg/mL, respectively.

FIGURE 1Quantitative analysis of hydroxychavicol in PB. (A) Standard graph of five different hydroxychavicol standards. (B) HPLC chromatogram for hydroxychavicol standard and PB chloroform extract. HPLC chromatogram profiling of hydroxychavicol (HC) in PB‐M and PB‐C at 280‐nm detection. (C) HC in PB‐M with a concentration of 1.0 mg/mL and (D) HC in PB‐C with a concentration of 1.0 mg/mL. HC peaks were detected at a retention time of 4.00–4.16 min.

**Figure:**
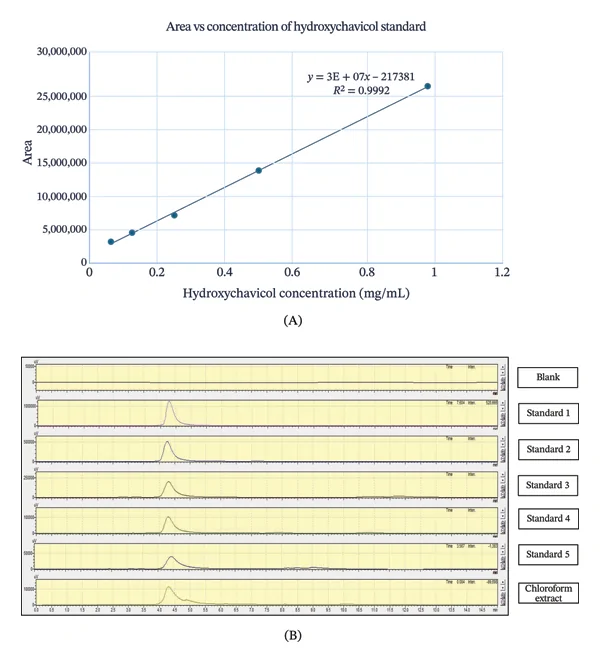

**Figure:**
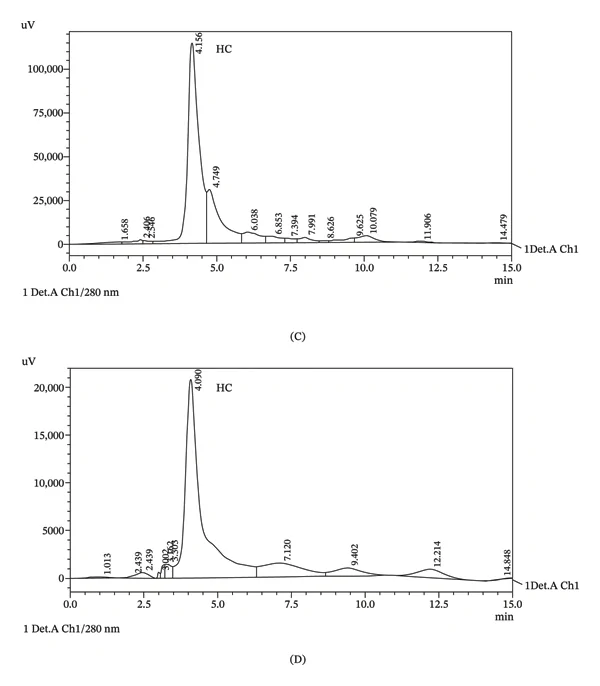

### 3.2. PB‐M and PB‐C Dose Dependently Exert Cytotoxic Effects on HUVEC

HUVEC treated with PB‐M at concentrations up to 100 µg/mL showed normal cobblestone morphologies. HUVEC displayed characteristics of cell death, such as cell shrinkage following PB‐M treatment at the concentration 125 μg/mL (Figure 2A). Correspondingly, HUVEC viability percentage displayed a concentration‐dependent decline in HUVEC viability with increasing concentrations of PB‐C (Figure 2B). The obtained IC_50_ value derived from the MTT assay for HUVEC treated with PB‐M was 128.9 µg/mL (Figure 2C).

FIGURE 2The effects of PB methanol extract (PB‐M) and PB chloroform extract (PB‐C) treatment on HUVEC. The effect of PB‐M extracts (1, 50, 100, 125, and 150 µg/mL) on (A) HUVEC morphologies, (B) the percentage of cell viability of HUVEC, and (C) IC_50_ value. The effect of PB‐C extracts 1, 10, 20, 50, and 100 µg/mL on (D) HUVEC morphologies, (E) the percentage of cell viability of HUVEC, and (F) IC_50_ value. Bars represent the mean ± standard error of the mean (SEM), *p* < 0.0001 (^∗∗∗∗^).

**Figure:**
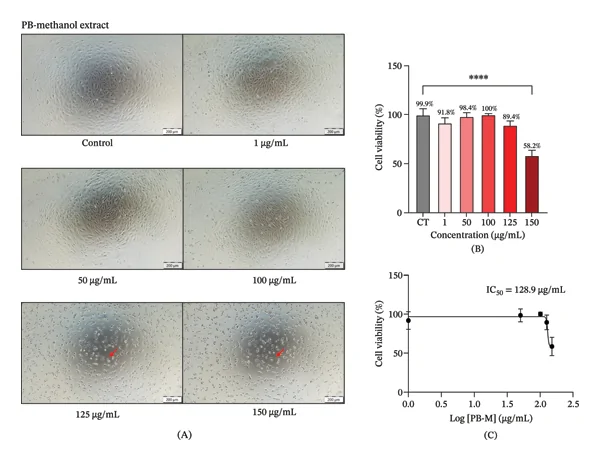

**Figure:**
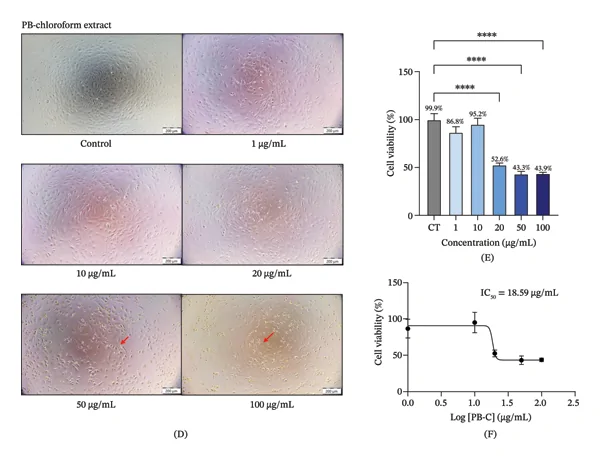

HUVEC exposed to PB‐C at concentrations of 50 μg/mL and above exhibited pronounced morphological changes, including cell rounding, elongation, and loss of cell‐to‐cell contact, indicative of cytotoxicity and reduced cell viability (Figure 2D). Correspondingly, quantitative analysis of cell viability revealed a concentration‐dependent reduction in HUVEC viability following PB‐C treatment (Figure 2E). The IC_50_ value for PB‐C on HUVEC was determined as 18.59 µg/mL (Figure 2F). Taking the results together, the concentrations tested for subsequent experiments were chosen to be below 100 and 20 µg/mL for PB‐M and PB‐C, respectively.

### 3.3. PB‐M and PB‐C Dose Dependently Reduce Expression of Adhesion Molecules in TNF‐α‐Stimulated HUVEC

To establish a robust disease control, TNF‐α concentrations ranging between 10 and 100 ng/mL, based on previous studies [28], were first tested. It was found that exposure to 100 ng/mL of TNF‐α resulted in the most pronounced expression of E‐selectin in HUVEC (Figure 3A). Therefore, 100 ng/mL was chosen as the standard concentration of TNF‐α for HUVEC activation in subsequent experiments.

**FIGURE 3:**
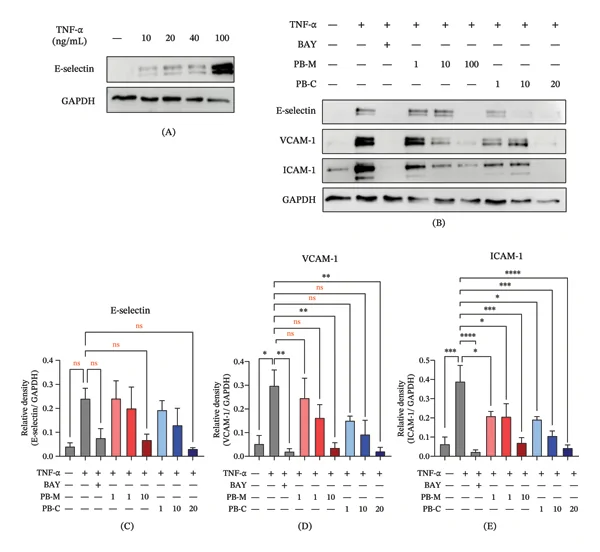
The effects of TNF‐α, PB‐M, and PB‐C treatments on the expression of adhesion molecules. The effect of (A) TNF‐α on the E‐selectin and GAPDH expression, (B) the effects of PB‐M and PB‐C treatment on the expression of adhesion molecules, E‐selectin, ICAM‐1, and VCAM‐1, and the densitometric analyses of (C) E‐selectin, (D) VCAM‐1, and (E) ICAM‐1 relative to GAPDH normalization. Bars represent the mean ± standard error of the mean (SEM), ns, not significant; *p* < 0.05 (^∗^), *p* < 0.01 (^∗^^∗^), *p* < 0.001 (^∗^^∗^^∗^), and *p* < 0.0001 (^∗^^∗^^∗^^∗^).

Expectedly, the stimulation of TNF‐α resulted in the pronounced expression of E‐selectin, VCAM‐1, and ICAM‐1 compared to the unstimulated control. Representative Western blot images demonstrated treatment with 100 μg/mL PB‐M and 10 μg/mL PB‐C resulted in a complete inhibition of E‐selectin, a result comparable to that of BAY 11‐7082, a standard NF‐κB inhibitor (Figure 3B). On the other hand, while a dose‐dependent reduction of VCAM‐1 and ICAM‐1 expression was observed in both extracts, a complete inhibition of the two adhesion molecules was only achieved following treatment with 100 μg/mL PB‐M and 20 μg/mL PB‐C (Figure 3B).

Densitometric analyses of E‐selectin, VCAM‐1, and ICAM‐1 bands from PB extract–treated HUVEC, compared with their respective controls, showed results consistent with the appearance of the corresponding western blot bands (Figure 3C–E). Densitometric analysis of E‐selectin revealed that PB‐M and PB‐C concentration dependently reduced E‐selectin expression although the reduction was not statistically significant. Notably, PB‐C at the concentration of 20 μg/mL shows a lower E‐selectin expression compared to 100 μg/mL PB‐M (Figure 3C). Similarly, VCAM‐1 expression exhibited a concentration‐dependent reduction following treatment with PB‐M and PB‐C, with significant suppression observed only at the highest concentration of each extract (PB‐M: 100 μg/mL and PB‐C: 20 μg/mL) (Figure 3D). Treatment of PB‐M and PB‐C at all concentrations studied significantly reduced the levels of ICAM‐1, as evidenced by the densitometric analysis (Figure 3E).

### 3.4. High Concentration of PB‐M Suppressed TNF‐α‐Induced IL‐6, IL‐8, and MCP‐1, Yet PB‐C Exhibited Cytokine‐Selective Suppression

As expected, IL‐6, IL‐8, and MCP‐1 levels in HUVEC were elevated following TNF‐α exposure, and treatment with the standard drug significantly suppressed IL‐6, IL‐8, and MCP‐1 expressions. IL‐6 level was significantly reduced following PB‐M treatment at 100 μg/mL, whereas PB‐C did not significantly suppress IL‐6 across all concentrations studied (Figure 4A). PB‐M treatment at the concentrations 10 and 100 μg/mL significantly reduced the level of IL‐8 relative to the TNF‐α‐stimulated control. Similarly, PB‐C treatment reduced IL‐8 level, with significant suppression observed at 10 μg/mL (Figure 4B). Both PB‐M and PB‐C displayed significant suppression of MCP‐1 at the highest concentrations, 100 and 20 μg/mL, respectively.

**FIGURE 4:**
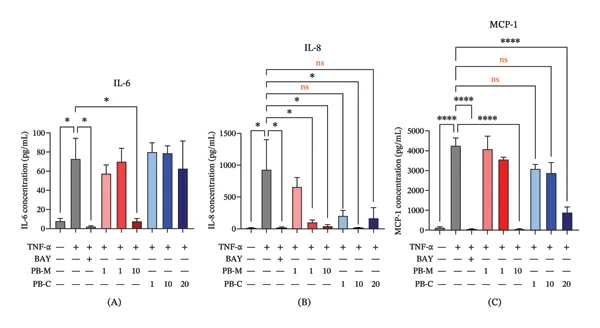
The expression of cytokines, IL‐6, IL‐8, and MCP‐1 followed by extract‐treated HUVEC. Quantitative analyses of (A) IL‐6, (B) IL‐8, and (C) MCP‐1 upon PB‐M and PB‐C treatments. Bars represent the mean ± standard error of the mean (SEM), ns, not significant; *p* < 0.05 (^∗^), *p* < 0.0001 (^∗∗∗∗^).

## 4. Discussion

In recent years, increasing evidence has demonstrated that PB‐derived extracts and polyphenols possess diverse pharmacological activities, with anti‐inflammatory effects being among the most extensively investigated [29, 30]. In this study, PB leaves were extracted using the maceration technique with two different solvent polarities, chloroform and methanol. Extractive yield is directly impacted by the choice of extraction technique, solvent, and efficiency. The higher yield of PB‐M over PB‐C likely stems from methanol’s polar protic nature and high dielectric constant, which effectively extract a wide array of polar phytochemicals, including glucosides and organic acids [25, 31]. The abundance of these primary and secondary metabolites substantially added to the dry mass of the crude extract, resulting in a higher overall extraction yield. Similar trends in the percentage extractive yield results were also reported by Madhumita et al. (2020) [32].

Based on the HPLC results, hydroxychavicol was detected in both PB extracts, with higher levels in PB‐C. Hydroxychavicol is a semipolar polyphenol by nature, stemming from its hydrophobic benzene ring and polar hydroxyl groups [33], favoring its solubility into semipolar solvents such as chloroform compared to highly polar methanol. This observation can be explained by the principle of “like dissolves like,” whereby solvents preferentially dissolve compounds with similar polarities [24]. Nonetheless, depending on the extraction conditions, compounds with semipolarity may partition into both solvent extract [34]. As a result, despite a lower overall yield of PB‐C, its hydroxychavicol content was higher than that of PB‐M. This observation was supported by a previous study of Zamakhshshari (2021), which demonstrated that hydroxychavicol content in the chloroform extract is higher than that in the methanol extract with the ratio of 66.73%–33.47% from 10 mg of the extract, respectively [25].

The vascular endothelium is essential for maintaining hemostasis and vascular health, serving as a semipermeable barrier that regulates the exchange of fluids, nutrients, and metabolites. High‐density lipoprotein maintains endothelial cells in a dormant condition [35]. The overexpression of adhesion molecules represents one of the earliest events of the pathogenesis of atherosclerosis [36]. Among adhesion molecules, VCAM‐1 and ICAM‐1 are essential for robust leukocyte attachment to endothelial cells [14, 37], whilst E‐selectin supports the initial recruitment of monocytes and their binding to expressed ligands during inflammation [38].

In this study, both PB‐M and PB‐C exhibit a dose‐dependent inhibition of the endothelial adhesion molecules, i.e., E‐selectin, VCAM‐1, and ICAM‐1 (Figure 5). Furthermore, the cytotoxicity findings revealed that PB‐C exhibited approximately 6‐fold higher cytotoxicity than PB‐M after the treatment of HUVEC. However, a lower cytotoxicity concentration (high IC_50_) is preferable, as it implies that the extracts is effective while remaining nontoxic, thereby reducing the risk of adverse effects on normal cells [39]. Nevertheless, PB‐C suppressive effects on the expression of TNF‐α‐induced adhesion molecules were significantly more pronounced compared to that of PB‐M treatment. This enhanced anti‐inflammatory activity of PB‐C may be due to its higher hydroxychavicol content. Collectively, this finding shows that PB compounds, whether polar or semipolar, have anti‐inflammatory properties, warranting further investigation as a potential therapy to mitigate endothelial dysfunction in the development of atherosclerosis.

**FIGURE 5:**
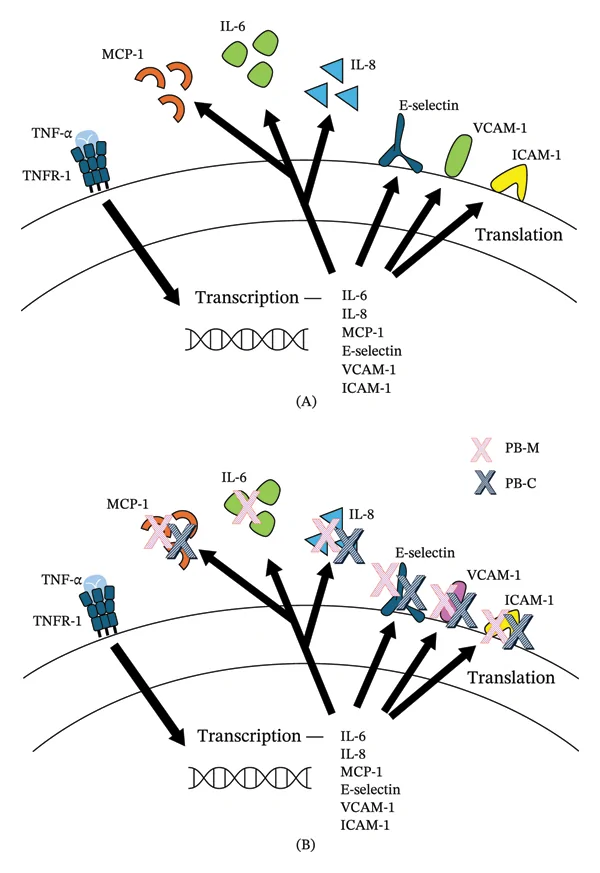
(A) TNF‐α signaling pathway induces the transcription and translation of proinflammatory adhesion molecules and cytokines. (B) Proinflammatory adhesion molecules and cytokines were reduced with the treatment of PB‐M and PB‐C.

Among the various compounds present in PB‐M and PB‐C extracts, hydroxychavicol can be commonly isolated from either extract [25, 40] and stands out for its notable anti‐inflammatory properties [41, 42]. Hydroxychavicol abundance is in agreement with the findings of T. Singh et al. (2023) [41], who reported that hydroxychavicol is the predominant phytochemical of PB, accounting for approximately 53% across all extracts. Hydroxychavicol is a potent inhibitor of COX‐1 and COX‐2 enzymes, important for prostaglandin synthesis, which is important in inflammation [43]. Furthermore, hydroxychavicol has been reported for its ability to inhibit the IκB and p65 phosphorylation and to prevent the induction of NF‐κB gene expression in the RAW 264.7 mouse macrophage cell line [29]. As a previous study has revealed that PB reduced the expression of adhesion molecules in macrophage RAW264.7 via downregulation of NF‐κB signaling pathway [23], it is plausible that the inhibition of adhesion molecules in HUVEC, as observed in this study, may be attributed to the suppression of the NF‐κB. Nevertheless, the hypothesis must be further investigated by, e.g., performing p65 nuclear translocation immunostaining or measurement of IκB or phospho‐p65 protein [44].

In atherosclerosis, the formation of foam cells that contributed to the atherosclerosis plaque instability occurs when macrophages phagocytose oxidized LDL [45, 46]. The recruited monocytes, by cell adhesion molecules, transdifferentiate into macrophages, acquiring new functional phenotypes. The activated endothelial cells secrete proinflammatory cytokines and chemokines, including IL‐6, IL‐8, and MCP‐1 [47]. In our study, we showed that PB‐M consistently inhibited IL‐6, IL‐8, and MCP‐1, prominently at the highest concentration (100 μg/mL) studied. PB‐C selectively suppressed IL‐8 and MCP‐1, but not IL‐6, even at the highest dose (20 μg/mL). Phytochemical‐rich plant fractions have been previously shown to exert significant anti‐inflammatory effects by inhibiting proinflammatory cytokines, including TNF‐α, and IFN‐γ in in vivo and in vitro models, supporting the notion that natural products can effectively modulate inflammatory pathways [48]. Additionally, emerging evidence indicates that natural products may target novel inflammatory targets, such as peptidyl‐arginine deiminases (PADs), further expanding their therapeutic potential [49]. Taken together, these findings, along with our results, suggest that the plant extracts, such as those investigated in this study, can effectively attenuate TNF‐α‐induced endothelial inflammatory responses, supporting their relevance as modulators of vascular inflammation.

Although the most reported transcriptional factor in the upregulation of IL‐6, IL‐8, and MCP‐1 is NF‐κB [50], it is suggested that there may be additional activated transcriptional factor exclusively for IL‐6 level in TNF‐α‐activated HUVEC. This is supported by the findings of Damasceno et al. (2021) [51], where TNF‐α was identified as one of the important cytokines in the upregulation of IL‐6 level through the activation of transcription factor NF‐IL6. Therefore, it is hypothesized that compound(s) in PB‐C might not target the additional transcription factor involved in the upregulation of IL‐6, such as NF‐IL6, although further investigation should be carried out to confirm this.

Although PB‐C demonstrated greater suppression of adhesion molecules, this effect should be interpreted alongside its cytotoxicity profile. Although PB‐C produced greater suppression of certain adhesion molecules, PB‐C exhibited a substantially lower cytotoxicity IC_50_ than PB‐M (18.59 versus 128.9 µg/mL, respectively), indicating greater cytotoxic potential and a narrower concentration range for biological activity. Therefore, the stronger inhibition observed for some inflammatory markers should not be interpreted as evidence of superior overall potency or therapeutic potential. In contrast, PB‐M exhibited a comparatively wider safety margin and broader suppression of the inflammatory mediators evaluated. PB‐C also displayed a more selective cytokine‐modulating profile, including the absence of significant IL‐6 suppression. Given the higher hydroxychavicol content of PB‐C, these differences further suggest that the biological effects of the crude extracts cannot be attributed solely to hydroxychavicol and may reflect differences in their overall phytochemical composition. Further studies defining the therapeutic window and characterizing the constituents responsible for both anti‐inflammatory activity and cytotoxicity are warranted. Nevertheless, since PB‐C is richer in hydroxychavicol content relative to PB‐M, IL‐6 signaling might be targeted by compounds that are more prominent in PB‐M, less in PB‐C. Several compounds that can be found in the PB methanol extract compared to the chloroform extract due to their polarity differences include syringic acid, rutin, apigenin, and eugenol [40]. Each of these compounds is known to downregulate IL‐6 in various in vivo and in vitro inflammatory models [52–55]. Taken together, the results suggest a significant role of hydroxychavicol in the suppression of TNF‐α‐induced adhesion molecules and cytokines in HUVEC.

While this study provides meaningful information on HUVEC molecular responses to the extract treatment in attenuating adhesion molecules and cytokines, it will be noteworthy to investigate the functional consequences of the suppression of cytokines and adhesion molecules. The inclusion of in vitro functional endothelial assays such as a permeability assay and monocyte adhesion assay would provide a more extensive evaluation of endothelial restoration following PB‐derived extract and compounds treatments [56]. Incorporating these functional endpoints will give a more comprehensive assessment of the vascular protective potential that are exhibited by PB‐derived extracts and compounds. Given the differential anti‐inflammatory responses observed between PB‐M and PB‐C, future studies beyond the evaluation of crude extracts and focusing on identifying specific compounds responsible for these effects are warranted. In particular, the interaction of phytochemicals present in the extracts should be investigated to determine whether the observed activities are mediated by individuals or by synergistic interactions between multiple constituents. Subsequently, promising single compounds or compound combination should be evaluated in appropriate animal models to establish their efficacy, safety, and dose–response under physiological conditions. These findings could provide a basis for optimizing clinically relevant doses and developing suitable formulations.

Additionally, future studies should focus on defining the molecular mechanisms underlying the anti‐inflammatory effects observed in the present study. Following the isolation and identification of bioactive constituents from PB extracts, their effects on NF‐κB signaling should be directly evaluated through assessment of IκBα degradation and/or phosphorylation, p65 phosphorylation, and p65 nuclear translocation. Such studies would establish whether the observed suppression of inflammatory mediators is directly associated with modulation of NF‐κB signaling and would enable mechanistic effects to be attributed to defined bioactive compounds rather than to the complex phytochemical composition of crude extracts.

## 5. Conclusion

Overall, our findings show that both PB‐M and PB‐C exert endothelial‐protective effects by attenuating TNF‐α‐induced inflammatory activation in HUVEC. Although PB‐C has a substantially higher hydroxychavicol levels and achieved its effects at lower doses, PB‐M consistently showed broader suppression across adhesion molecules and key proinflammatory cytokines, suggesting that additional bioactive constituents in PB‐M may act synergistically with hydroxychavicol. In contrast, PB‐C exhibited a more selective cytokine‐modulating profile, indicating differential mechanistic actions between the two extracts. These results highlight PB as a promising source of anti‐inflammatory agents and underscore the importance of extraction methods in shaping its bioactivity. Further studies are warranted to delineate the specific phytochemical interactions responsible for these distinct anti‐inflammatory responses and to explore their therapeutic relevance in vascular inflammatory disorders.

## Funding

This study was funded by Universiti Malaysia Sarawak, UNI/F07/TZC/85387/2024.

## Conflicts of Interest

The authors declare no conflicts of interest.

## Data Availability

The datasets generated and analyzed during the current study are available from the corresponding author upon reasonable request.
